# Functional Characterization of F3H Gene and Optimization of Dihydrokaempferol Biosynthesis in *Saccharomyces cerevisiae*

**DOI:** 10.3390/molecules29102196

**Published:** 2024-05-08

**Authors:** Qinyi Chen, Dandan Song, Xiaoyan Sun, Yulong Tian, Zecheng Yan, Ting Min, Hongxun Wang, Limei Wang

**Affiliations:** 1 College of Life Science and Technology, Wuhan Polytechnic University, Wuhan 430023, China; 18278911084@163.com (Q.C.); 13972457283@163.com (D.S.); sunxiaoywu@foxmail.com (X.S.); t15513387881@163.com (Y.T.); yanzechengyx@163.com (Z.Y.); wanghongxun7736@163.com (H.W.); 2 College of Food Science and Engineering, Wuhan Polytechnic University, Wuhan 430023, China; minting1323@163.com

**Keywords:** TbF3H, molecular cloning, function analysis, promoter adjustment

## Abstract

The 1092 bp F3H gene from *Trapa bispinosa* Roxb., which was named TbF3H, was cloned and it encodes 363 amino acids. Bioinformatic and phylogenetic tree analyses revealed the high homology of TbF3H with flavanone 3-hydroxylase from other plants. A functional analysis showed that TbF3H of *Trapa bispinosa* Roxb. encoded a functional flavanone 3-hydroxylase; it catalyzed the formation of dihydrokaempferol (DHK) from naringenin in *S. cerevisiae*. The promoter strengths were compared by fluorescence microscopy and flow cytometry detection of the fluorescence intensity of the reporter genes initiated by each constitutive promoter (FITC), and DHK production reached 216.7 mg/L by the promoter adjustment strategy and the optimization of fermentation conditions. The results presented in this study will contribute to elucidating DHK biosynthesis in *Trapa bispinosa* Roxb.

## 1. Introduction

*Trapa bispinosa* (*Trapa bispinosa* Roxb.) is an annual aquatic herbaceous floating plant of the genus Trapa in the family Rhodophyta of the order Myrtaceae, found mainly in tropical and temperate regions [1,2]. Rhododendron is native to Europe and Asia, and in China it is mainly distributed in the middle and lower reaches of the Yangtze River [3], one of the aquatic specialties of Hubei Province, which has high economic value. Some studies have shown that *Trapa bispinosa* shells and leaves can be used to treat common diseases such as gastric ulcers and esophageal cancers, and they also have antibacterial [4], antitumor [5], et cetera, effects. The shells and leaves are rich in a variety of biologically active compounds such as phenols and flavonoids, which have been extensively studied [6]. The shells contain many flavonoids, such as naringenin, dihydromountaineol, delphinidin and delphinidin-3-O-glucoside [7]. These flavonoids have specific physiological antioxidant [8], anti-inflammatory [9], and anticancer [10] properties.

Dihydrokaempferol (DHK), isolated mainly from citrus and zinnia, is a widely used natural pigment and flavonoid with various pharmacological activities. Studies have shown that DHK can ameliorate severe acute pancreatitis through the Keap1/Nrf2 pathway [11]. DHK also attenuates LPS-induced inflammation and apoptosis in Wl38 cells [12] and has apoptotic effects on synoviocytes. In addition, DHK detoxicates CCl4 by inhibiting RARP-1, thereby affecting multiple downstream pathways and cytokine-induced liver fibrosis [13]. DHK induces apoptosis and inhibits Bcl-2 and Bcl-xL expression and could be a good candidate for a new anti-arthritic drug [14].

Flavanone-3-hydroxylase (F3H) was first discovered in Antirrhinum majus, and homologous genes were subsequently found in other species, such as Arabidopsis thaliana [15], wolfberry [16], tea [17], and soybean [18]. F3H is a member of the 2-oxoglutarate-dependent dioxygenase family and is a key enzyme in the flavonoid biosynthetic pathway. In plants, F3H catalyzes the 3-hydroxylation of flavanols to form dihydroflavonols, and it more commonly catalyzes naringenin to produce dihydrocannabinol, which is a precursor substance of flavonols and anthocyanins. Numerous studies have shown that F3H is a key gene in the regulation of flavonoid accumulation at the flavonol and anthocyanin branches [19,20]. Furthermore, F3H not only regulates flavonoid composition but also plays an important role in plant stress tolerance, and its expression is induced with increases in flavonoid content under abiotic stress, which helps to protect the plant from oxidative damage [21,22,23].

So far, most studies have focused on the isolation and pharmacological effects of this active monomer compound, and the biosynthetic mechanism of its active component in *Trapa bispinosa* and the clonal expression analysis and functional characterization of the F3H gene have not yet been reported. In this study, the F3H gene was annotated and screened using transcriptome technology, and the TbF3H gene sequence was obtained. After codon optimization, a recombinant expression vector was constructed and expressed in Saccharomyces cerevisiae, and its enzymatic activity was determined. The promoter strengths were compared by fluorescence microscopy and flow cytometry detection of the fluorescence intensity of the reporter genes was initiated by each constitutive promoter (FITC). Additionally, DHK production was improved by the promoter adjustment strategy and optimization of fermentation conditions. The molecular characterization and functional identification of the TbF3H gene proposed in this study will help us to elucidate the accumulation of flavonoids in lozenges.

## 2. Results

### 2.1. DHK Biosynthetic Pathway

**Figure 1:**
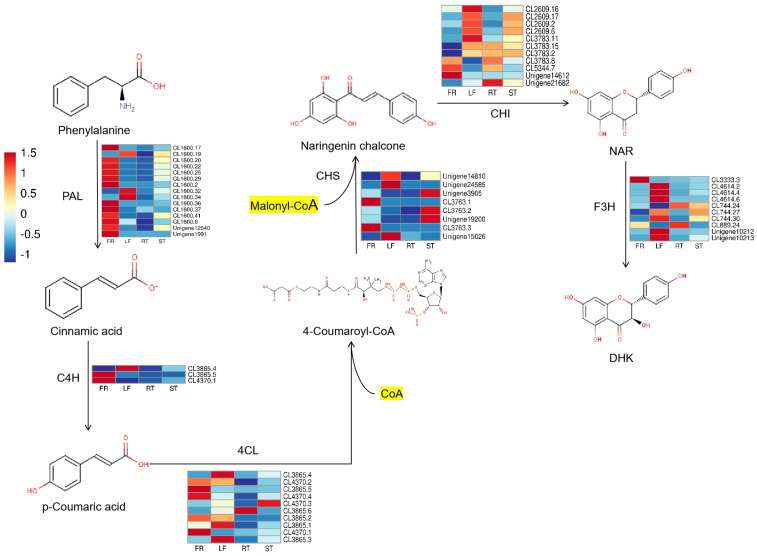
Dihydrokaempferol (DHK) biosynthesis pathway and the expression of related genes. The heatmaps next to these enzymes were drawn from the FPKM values of unigenes annotated as corresponding genes. The square on the heatmap represents one of the three repeats of a gene. The color indicates the relative expression intensity of the gene; the higher the value, the higher the intensity of gene expression.

The DHK biosynthesis pathway begins with L-phenylalanine, which is catalyzed by phenylpropanoid-related core biosynthesis genes phenylalanine ammonia-lyase (PAL), cinnamic-4-hydroxylase (C4H), and 4-coumaryl-CoA ligase (4CL), which is a precursor for the biosynthesis of all phenolic secondary metabolites in higher plants. Coumaryl-CoA and malonyl-CoA are catalyzed by chalcone synthase (CHS) to form chalcone, followed by the isomerization of chalcone isomerase (CHI) to form Naringenin (NAR), an important backbone of flavonoids. DHK is subsequently generated by NAR catalyzed by Flavanone-3-hydroxylase (F3H). In this study, a total of 57 single genes belonging to the DHK biosynthesis pathway were identified in combination with transcriptome data from *Trapa bispinosa* Roxb., and the expression levels of the genes were shown with heat maps (Figure 1).

### 2.2. Expression Level of TbF3H Gene in the Trapa bispinosa *Roxb*.

**Figure 2:**
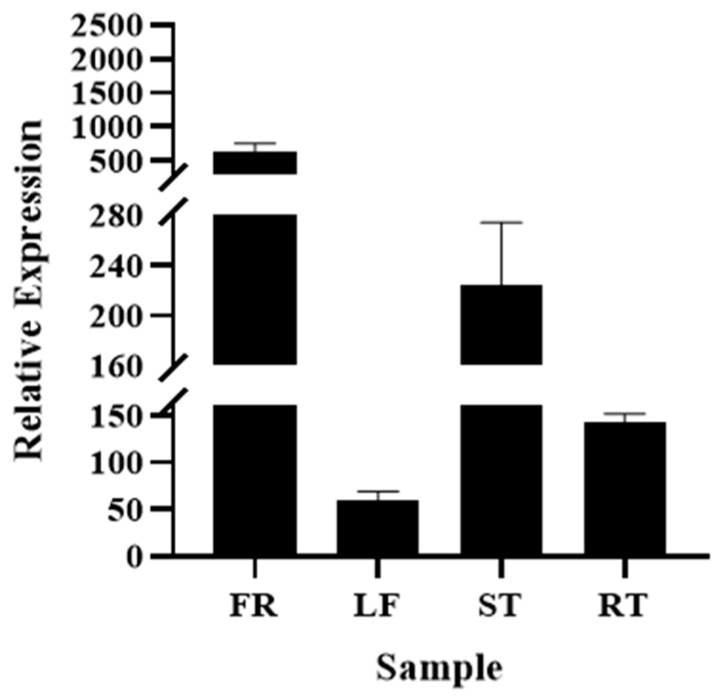
Expression level of TbF3H gene. Shell (FR), leaf (LF), stem (ST), and root (RT).

Quantitative real-time PCR (qRT-PCR) was used to detect the expression level of the TbF3H gene in four tissues: shell (FR), leaf (LF), stem (ST), and root (RT) (Figure 2). Apparent differences in transcription levels were observed in different parts, the highest being FR expression, and ST expression being the second highest. In contrast, the RT and LF yielded relatively low expressions.

### 2.3. The TbF3H Gene Structure Analyses

**Figure 3:**
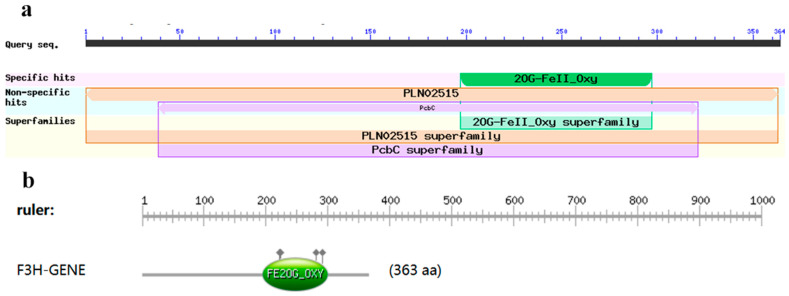
Conserved domains analyses of TbF3H protein. (**a**): Results of structural domain analysis of TbF3H protein. (**b**): Interval of the structural domain of 2OG-Fe(II) oxygenase.

The predicted results of primary protein structure showed that the TbF3H gene encoded a total of 363 amino acids, which corresponds to a molecular mass of 40.8 kDa, with a theoretical pI of 5.46 based on the ProtParam online analysis software available online. A Conserved Domains (CDs) search predicted that the TbF3H protein belongs to the PLN02515 superfamily (cl31892), with a structural domain interval ranging from amino acids 1 to 363, and amino acids 2 to 297 of the 2OG-Fe(II) oxygenase (pfam03171) superfamily (Figure 3).

**Figure 4:**
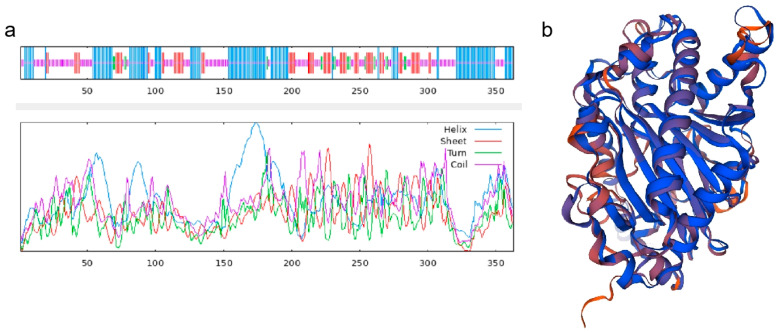
TbF3H protein sequence secondary structures (**a**) and tertiary structure prediction (**b**).

The secondary structure of the TbF3H protein sequence was analyzed using SOPMA. The secondary structure of the TbF3H protein is shown in Figure 4a, with 37.47% α-helices (blue), 19.28% extended strands (red), 5.23% β-turns (green), and 38.02% irregular curls (purple). SWISS-MODEL modeling of sequence homology of the TbF3H protein showed that the template used was 2-oxoglutarate-dependent dioxygenase from Punica granatum, and the molecular name of the template was A0A218XVH5.1.A, which had 90.36% similarity to the TbF3H protein (Figure 4b).

### 2.4. Comparison of Homology between TbF3H Protein and Phylogenetic Tree Construction

**Figure 5:**
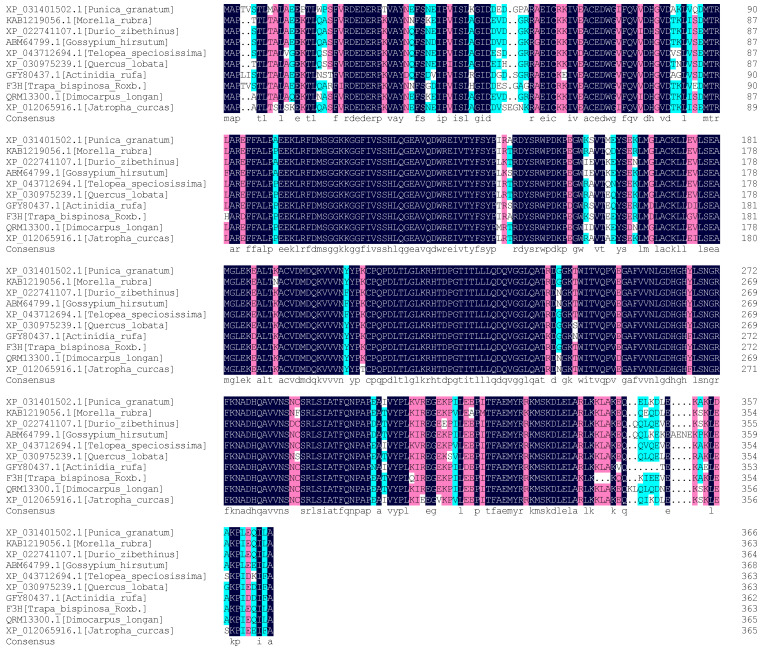
Comparison of TbF3H amino acid sequences obtained from GenBank. Black is a highly conserved sequence. In red, more than 75% are conserved sequences. Blue is more than 50% are conservative sequences.

In the homology analysis of the TbF3H protein sequence, the compared species included Punica granatum, Morella rubra, Durio zibethinus, Gossypium hirsutum, Telopea speciosissima, Quercus lobata, Actinidia rufa, Dimocarpus longan, and Jatropha curcas. The results showed a similarity of 91.21% between the protein and the compared sequence (Figure 5).

**Figure 6:**
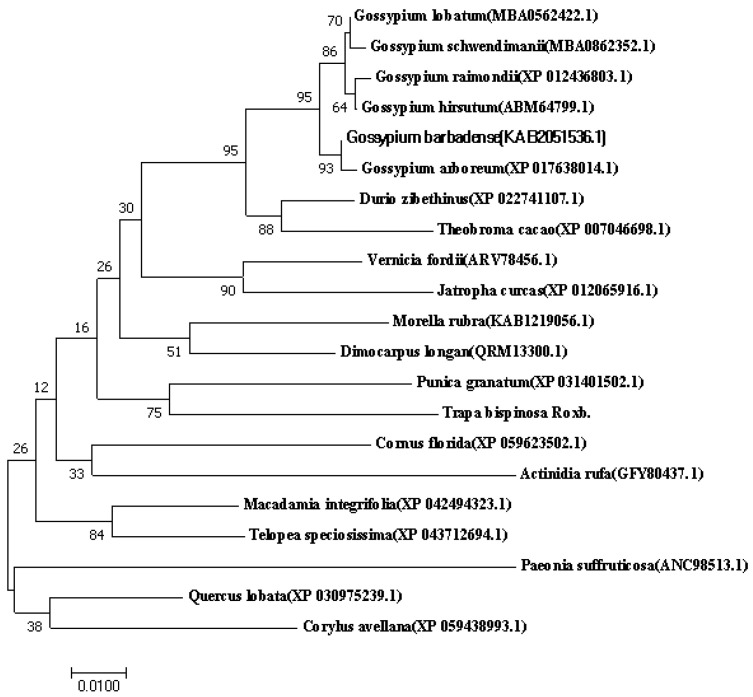
Phylogenetic analysis of TbF3H based on amino acid sequences. The numbers in parentheses represent the accession numbers in the NCBI for the corresponding protein sequences. Line segment 0.01 represents evolutionary distance units.

The TbF3H protein sequences were aligned by NACI Blast, and the F3H amino acid sequences of 20 plants were screened from the GenBank alignment database, and then the phylogenetic trees of F3H proteins of different species were constructed by MEGA-X7 software. As shown in Figure 6, *Trapa bispinosa* Roxb. was most closely related to *Punica granatum* L.

### 2.5. TbF3H Gene Cloning and Vector Construction

**Figure 7:**
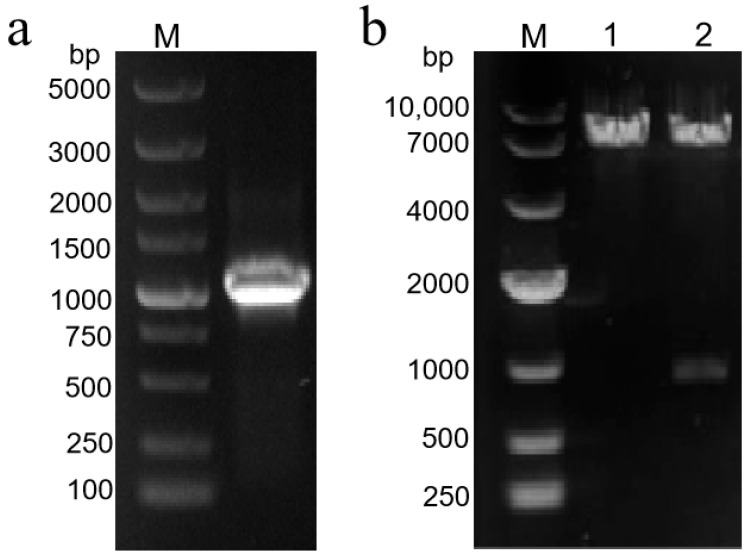
Isolation and identification of F3H gene from *Trapa bispinosa* Roxb. (**a**) TbF3H sequence amplification electrophoresis. (**b**) Electrophoresis pattern of double-enzyme-digested recombinant plasmid. 1: Results of double digestion of pESC-URA empty vector; 2: results of double digestion of pESC-URA-TbF3H recombinant plasmid.

PCR amplification of the TbF3H gene from *Trapa bispinosa* Roxb. shells (Figure 7a) and the target gene was integrated into the pESC-URA yeast expression vector. The plasmid was transformed to the Escherichia coli receptor cells, positive clones were screened by colony PCR, and the plasmid was extracted for double enzyme digestion and sent to sequencing for verification, and the recombinant plasmid pESC-URA-TbF3H was obtained after digestion and verification (Figure 7b).

### 2.6. Determination of Enzyme Activity of TbF3H Protein in S. cerevisiae

**Figure 8:**
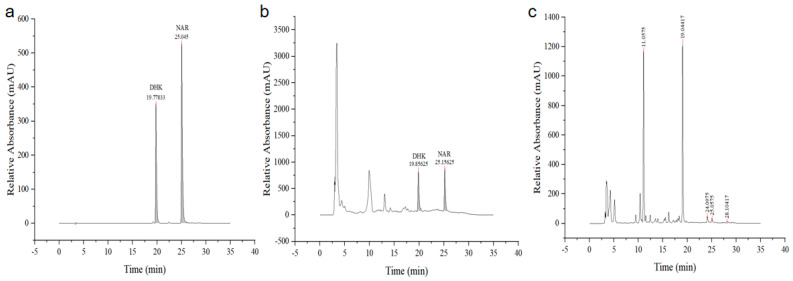
Determination of recombinant F3H enzyme activity using HPLC. (**a**) NAR and DHK standard; (**b**) DHK experimental group; and (**c**) black group.

The substrate NAR was added after 72 h of shake flask fermentation in Saccharomyces cerevisiae, and the formation of DHK was detected by HPLC. As shown in Figure 8, the retention time of the DHK standard was 19.66 min, and the signal peak occurred in the experimental group at 19.73, which was similar to that of the DHK standard, while no signal peak appeared in the blank group. It was initially concluded that TbF3H protein could catalyze the formation of DHK from NAR.

**Figure 9:**
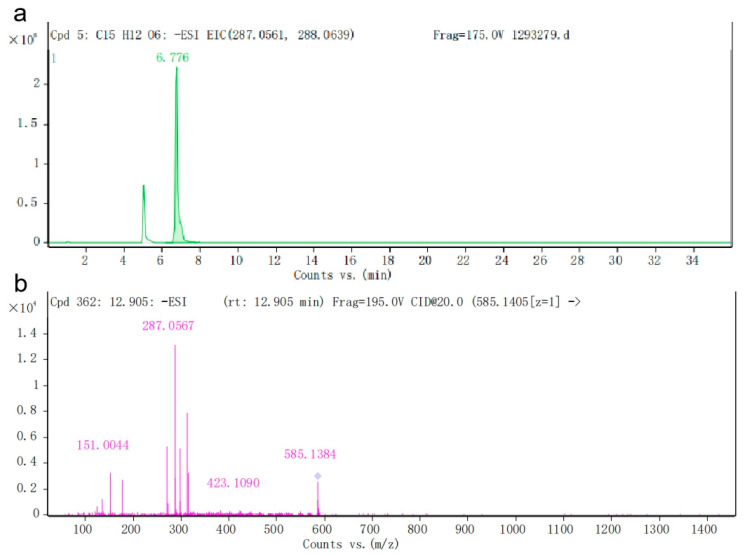
Determination of recombinant F3H enzyme activity using LC-MS. (**a**) DHK peaking time; (**b**) DHK total ion flow diagram.

To further determine the product catalyzed by the TbF3H protein in yeast fermentation, Liquid Chromatography–Mass Spectrometry (LC-MS) was used to detect the product composition in the fermentation samples (Figure 9). The mass spectrometry results showed the presence of DHK characteristic ion fragments in the fermentation sample with m/z of 287.0578. The molecular formula of the sample was consistent with that of the DHK standard, which was C_15_H_12_O_6_, and the results were in agreement with the negative ion scanning results of the standard.

### 2.7. Verification of Promoter Strength with EGFP through Flow Cytometer 

**Figure 10:**
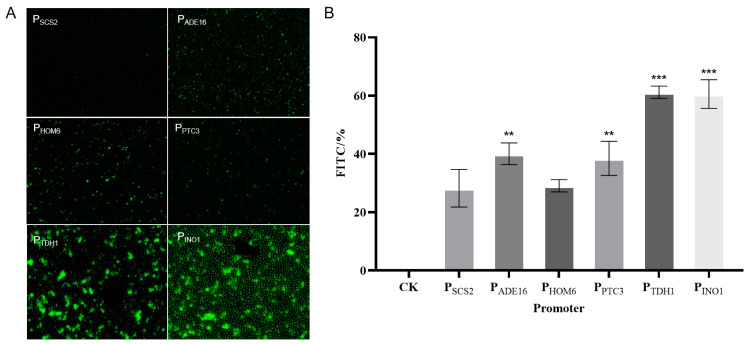
Comparison of fluorescence intensity of different constitutive promoters. (**A**): Fluorescence microscopy results. (**B**): Flow cytometry results. ** means that the results are generally significant. *** means that the results are highly significant.

The expression of EGFP was detected by a fluorescent inverted microscope as a way to verify promoter expression (Figure 10A). The transcription activity of six promoters was verified on the basis of EGFP fluorescence strength by a flow cytometer. The mean values of fluorescence intensity were used to represent promoter transcription activity. An ideal promoter should have strong EGFP fluorescence intensity. The transcription activities of the six promoters were compared using the EGFP-free vector as a blank control (Figure 10B). The results showed that P_TDH1_ and P_INO1_ were strong constitutive promoters, as detected by flow cytometry and fluorescence microscopy.

### 2.8. Enhancement of DHK Production by Promoter Adjustment

**Figure 11:**
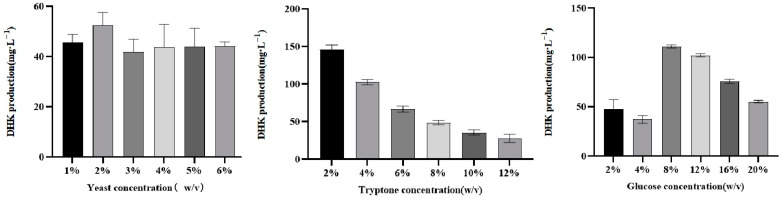
Production of DHK after promoter adjustment. After promoter adjustment, seven preferable strains were verified through HPLC from 250 mL shake flasks with 25 mL YPD liquid medium.

To further optimize the expression of TbF3H, a series of promoters with different strengths (two strong promoters: P_PTC3_ and P_INO1_; two medium-strength promoters: P_HOM6_ and P_TDH1_; and two weak promoters: P_ADE16_ and P_SCS2_) were selected from the Saccharomyces cerevisiae promoter library [24] for combinatorial analyses, and the results are shown in Figure 11. As can be seen from the figure, the DHK product of the galactose promoter P_GAL1_ was only 27.75 mg/L. After replacing the constitutive promoters with different strengths through the promoter adjustment strategy, the DHK product increased significantly. Among them, the DHK product of the strong promoter P_PTC3_ reached 60.0 mg/L, which was a 2.2-fold increase in DHK production compared with the galactose promoter.

### 2.9. Fermentation Production of Dihydrocamptothecin under Different Fermentation Conditions

The PPTC3 promoter fermentation strain was selected for the optimization of fermentation conditions to explore the effects of different fermentation conditions on the products by medium concentration and cofactor addition, and to detect the production of dihydrosorbinol by HPLC.

**Figure 12:**
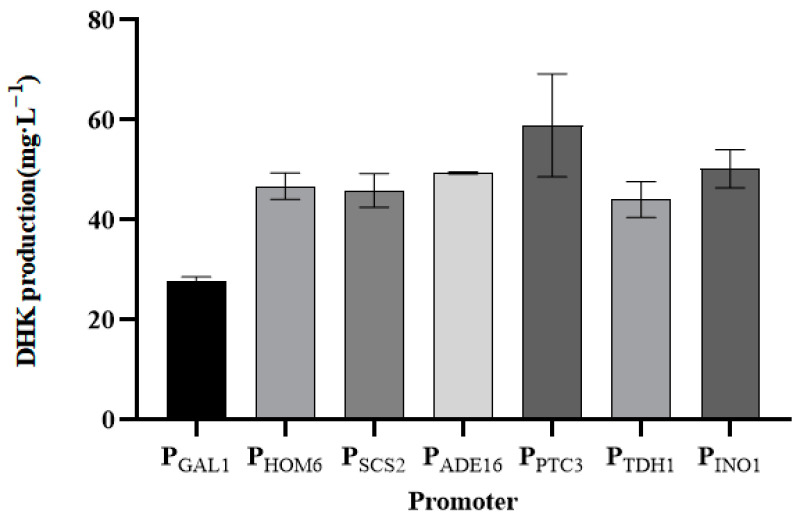
Variation in DHK content at different carbon and nitrogen source concentrations.

The first is the optimization of the concentration of each component of the fermentation medium YPD, keeping the inoculum amount consistent, controlling the concentration of each component of the medium for fermentation, and detecting the fermentation production of the product dihydrosorbinol. The results are shown in Figure 12. The highest DHK production of 146.08 mg/L was achieved when the concentration of Yeast was 1%, Tryptone was 2%, and Glucose was 8%. When the sugar concentration exceeded 8%, the content of DHK began to decrease, probably due to the high concentration of sugar in the medium, which is highly permeable and would be consistent with the growth and reproduction of Saccharomyces cerevisiae.

**Figure 13:**
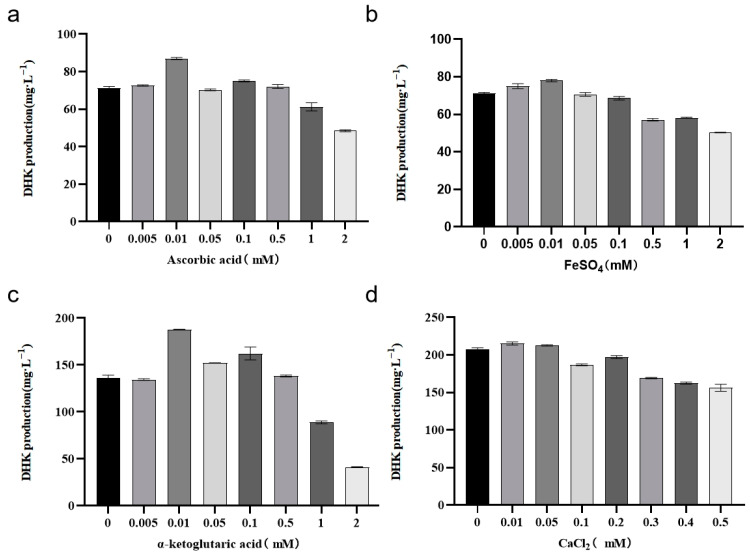
The effect of cofactors on DHK production. (**a**): Diagram of ascorbic acid fermentation results. (**b**): Graph of FeSO_4_ fermentation results. (**c**): Diagram of α-ketoglutaric acid fermentation results. (**d**): Diagram of CaCl_2_ fermentation results.

Then, fermentation experiments were carried out at the concentrations of the medium components determined above by adjusting the additions of cofactors, α-ketoglutaric acid, ascorbic acid, ferrous sulfate, and CaCl_2_, each with eight concentration gradients. The results are shown in Figure 13. Ascorbic acid addition at 0.01 mM had the best effect, the DHK yield was 87.6 mg/L, and a further increase in the concentration of ascorbic acid had no obvious effect on the DHK yield enhancement (Figure 13a). FeSO_4_ addition in the range of 0.005 mM to 0.01 mM had a better effect (Figure 13b). The best effect was achieved when α-ketoglutarate was added at 0.01 mM, and the DHK yield reached 188.04 mg/L. When the added amount was greater than 0.5 mM, the growth of the bacterium was seriously inhibited, which led to a serious decrease in the yield of DHK (Figure 13c). The added amount of CaCl_2_ was in the range of 0.01 mM~0.05 mM, and it was the most effective of the four cofactors for the enhancement of the yield of DHK. The yield of DHK was the highest, reaching 216.7 mg/L (Figure 13d).

## 3. Discussion

As an alien species, *Trapa bispinosa* Roxb. has been domesticated and cultivated in China [25]. Wuhan, China, is one of the cultivation bases of *Trapa bispinosa*, as the climate and environment are suitable for the growth and development of the plant [26]. Presently, research on *Trapa bispinosa* Roxb. is focusing on the extraction of starch materials [27,28,29], the pharmacological activity of flavonoids [30,31], etc. Recent studies have shown that there are a variety of biologically active compounds such as phenolic acids, flavonoids, terpenoids, etc., within the husk and leaves of the loosestrife, many of which have been shown to have good effects on antimicrobial and antitumor properties. DHK is a dihydroflavonol with a variety of pharmacological activities, such as anticancer and anti-inflammatory. At present, the research on DHK mainly focuses on its pharmacological activity, but the synthetic pathway of DHK in *Trapa bispinosa* Roxb. has not been elucidated. So, alongside cloning the F3H gene from *Trapa bispinosa* Roxb., a series of experiments were carried out on the biosynthesis of DHK.

In plants, F3H has an important role in the biosynthetic pathway of flavonoids. It catalyzes the 3-hydroxylation of Naringenin to form dihydrokaempferol, which is an intermediate for the biosynthesis of flavonols and anthocyanins. Previous studies have suggested that F3H plays an important role in the regulation of flavonol and anthocyanin accumulation in plants. In maize anthers, the expression of F3H was found to temporally coordinate with the appearance of flavonols [32]. In addition, F3H was also found to be a rate-limiting enzyme in anthocyanin biosynthesis. Recent studies have shown that the ethylene response factor ERF5 regulates anthocyanin biosynthesis in “Zijin” mulberry fruits by interacting with MYBA and F3H genes [33]. The high expression of F3H has led to the accumulation of anthocyanin in muscadine grapes and strawberry fruit [16,34]. 

It was found that the silencing of the apple MdF3H gene using antisense technology resulted in an increase in flavanones but a decrease in their downstream products compared to the wild type, suggesting that mutations in the F3H gene inhibit the conversion of flavanones to downstream metabolites. Jan et al. showed that overexpression of OsF3H gene in rice (*Oryza sativa* L.) significantly increased flavonoid biosynthesis [35]. Thus, repression or overexpression of the F3H gene leads directly to the down- or up-regulation of flavonoid metabolism synthesis. To date, although F3H has been cloned and characterized in several plant species, the research on F3H in *Trapa bispinosa* Roxb. is limited and its roles in the regulation of flavonoid accumulation in citrus fruit are still far from being elucidated.

This study explored *Trapa bispinosa* Roxb. The F3H gene was isolated and analyzed by bioinformatics. The CD search prediction showed that the TbF3H protein had a 2OG-Fe(II) oxygenase domain, amino acid sequence alignment showed that TbF3H was highly conserved in plant species, and phylogenetic tree analysis showed that TbF3H was the closest to guava. Enzyme function verification showed that the functional flavanone 3-hydroxylase encoded by TbF3H could catalyze the generation of DHK from NAR. The promoter strengths were compared by fluorescence microscopy and flow cytometry detection of the fluorescence intensity of the reporter genes initiated by each constitutive promoter (FITC), and DHK production was improved by the promoter adjustment strategy. The DHK product of the strong promoter PPTC3 reached 60.0 mg/L. The subsequent optimization of fermentation conditions resulted in a DHK production of 216.7 mg/L. This is the highest production of DHK that we currently know.

## 4. Materials and Methods

### 4.1. Plant Materials

In this study, the plant materials of *Trapa bispinosa* Roxb. were laboratory-preserved. The genetic screen and RNA-seq data analysis were based on a pre-laboratory study of the *Trapa bispinosa* Roxb. transcriptome [7].

### 4.2. RNA Extraction and TbF3H Enzyme Gene Cloning

Extraction of total RNA from *Trapa bispinosa* Roxb. was carried out using the Trizol method. Single-strand cDNA was synthesized using the HiScript III 1st Strand cDNA Synthesis Kit (+gDNA wiper) (Vazyme Bio, Nanjing, China). Specific primers were based on the TbF3H sequence information and designed using Snapgene, as shown in Table 1. The TbF3H gene was amplified using PCR with 2 × KeyPo Master Mix (Dye Plus) (Vazyme Bio, Nanjing, China), and an OMEGA PCR Gel Extraction Kit was used to extract the amplified product.

### 4.3. qRT-PCR Analysis

Shell (FR), leaf (LF), stem (ST), and root (RT) from the samples frozen at −80 °C, and the total RNA of the four samples was extracted using the Trizol method. Reverse transcription into cDNA was performed using the HiScript III 1st Strand cDNA Synthesis Kit (+gDNA wiper) (Vazyme Bio, Nanjing, China) as the template for qRT-PCR. Table 1 displays the primer sequences. The reaction systems were prepared according to TB Green^®^ Premix Ex TaqTM II (Takara Bio, Beijing, China). The relative gene expression level was calculated using the 2^−ΔΔCt^ method [36].

### 4.4. Bioinformatic Analysis of TbF3H Gene

The physicochemical characterization of F3H enzyme genes was performed using the online software Protparam. The conserved protein structural domains and their families and functional sites were analyzed by CD Search [37]. The protein secondary structure was analyzed using the software SOPMA, and the protein tertiary structure was modeled using SWISS-MODEL. The amino acid sequences of the encoded protein were compared using BLAST, and using DNAMAN and MEGA to perform homologous sequence analysis and build the phylogenetic tree.

### 4.5. Expression of Plasmids and Strain Construction

After the successful sequencing verification of the pET-28a plasmid, the target gene was sent to GenScript for codon optimization, and the optimized target fragment was integrated into the pESC-URA yeast expression vector. Six constitutive promoter expression vectors were constructed, and these kinds of plasmids were constructed using an easy-to-use one-step cloning kit (Vazyme Bio, Nanjing, China). The constructed plasmids were sequenced and verified, transformed into Saccharomyces cerevisiae BY4741 receptor cells, and screened for Saccharomyces cerevisiae strains on CM-URA-deficient and AmpR-resistant plates. The constructed plasmids and constitutive promoter specific primers are shown in Table 2.

### 4.6. Enzyme Activity Detection of TbF3H Protein

Culture conditions: All recombinant yeasts were grown on plates in nutrient-poor CM medium (6.7 g/L yeast nitrogen base, 20 g/L glucose, 0.83 g Dropout powder, and appropriate amino acids: 50 mg/L adenine, 50 mg/L leucine, 100 mg/L histidine, 100 mg/L tryptophan, and 50 mg/L uracil) for 2–4 days at 28 °C. Galactose promoter fermentation: Single colonies were inoculated in 25 mL PA bottles containing 5 mL CM medium at 230 rpm, activated at 28 °C for 24 h, and centrifuged at 4 °C for 5 min at 5000× *g*. The strains were resuspended in YPD culture medium containing 4% galactose at 230 rpm and 28 °C for 24 h. After the addition of 25 mg/mL of the substrate naringenin (methanol solubilized) at 28 °C, 230 rpm was used for further cultivation, then 230 rpm, and incubation was continued for 72 h. Constitutive promoter fermentation: Single colonies were inoculated in 25 mL PA flasks containing 5 mL CM medium at 220 rpm and activated for 24 h at 28 °C, centrifuged for 5 min at 4 °C and 5000× *g*, and then the strain was resuspended in 25 mL YPD culture medium containing 25 mg/mL naringenin as the substrate and incubation was continued for 72 h at 230 rpm and 28 °C.

Analytical methods: After fermentation, 800 μL of bacterial liquid was taken, and an equal volume of 1% hydrochloric acid methanol was added, vortexed, and mixed, and then extracted by ultrasonic extraction for 30 min, and centrifuged at 12,000× *g* for 5 min at 4 °C to separate the supernatant from the cell pellet. The supernatant was then passed through a 0.22 μm filtration membrane, and then the products were analyzed and detected by high-performance liquid chromatography (HPLC) [38]. The liquid-phase conditions and methods are described below.

HPLC: chromatographic column, Agilent C18 column; mobile phase, 0.5% acetic acid water (A) and 0.5% Formic acid methanol (B); injection volume 100 μL; flow rate 0.8 mL/min; column temperature 35 °C; detection wavelength 290 nm. Liquid-phase method: 0–20 min, 15–60% B; 20–25 min, 60% B; 25–35 min, 15% B. The liquid-phase diagram of standard DHK was compared with the experimental results to confirm product formation.

LC-MS: chromatographic column, waters BEH (C18 2.1 × 100 mm 1.7 um); mobile phase, 0.1% formic acid aqueous solution (A) and methanol (B). Gradient elution: 0~20 min, 85~40% A; 20~25 min, 40% A; 26 min, 85% A; 36 min, 85% A. Volume flow rate 0.3 mL/min; injection volume 5μL; column temperature, 35 °C.

Mass spectrometry conditions: negative ion scanning mode (ESI; m/z 50~1500); capillary voltage, 3.2 Kv; sheath gas temp 350 °C; sheath gas flow 12 L/min.

### 4.7. Optimization of Fermentation Conditions of Saccharomyces cerevisiae to Improve DHK Production

The addition of carbon and nitrogen sources to the yeast YPD fermentation medium was optimized to find out the optimal fermentation conditions suitable for the target strains. The optimized settings for media fermentation conditions are shown in Table 3.

In addition, the enzymes related to flavonoid synthesis require a variety of cofactors to exert their catalytic activity, commonly α-Ketoglutaric acid, ascorbic acid, Fe^2+^ and Ca^2+^, etc. Relying on only a small amount of cofactors in the medium may not be able to exert the maximum enzyme activity, so exogenously added cofactors need to be tested in order to find the optimal amount of cofactors to be added. The optimized settings for cofactor fermentation conditions are shown in Table 4.

## 5. Conclusions

In this study, the gene TbF3H was successfully cloned from *Trapa bispinosa* Roxb. After gene optimization, the nucleic acid and protein sequences were analyzed using bioinformatics and the phylogenetic tree and were placed into Escherichia coli. Then, Saccharomyces cerevisiae expression vectors were constructed. The HPLC results showed that TbF3H could catalyze NAR to produce DHK. After replacing the constitutive promoters with different strengths through the promoter adjustment strategy, the DHK product of the strong promoter P_PTC3_ reached 60 mg/L. The subsequent optimization of fermentation conditions resulted in a DHK production of 216.7 mg/L. This study confirmed the catalytic role of TbF3H in DHK biosynthesis and contributed to a better understanding of flavonoid biosynthesis in *Trapa bispinosa* Roxb.

## Figures and Tables

**Table 1:** Primer sequences.

Primer Name	Sequence (5′-3′)
TbF3H_F	TGGACAGCAAATGGGTCGCGGATCCATGGCTCCCACGGTCTCGAC
TbF3H_R	CGAGTGCGGCCGCAAGCTTGTCGACTTAAGCTAAGATCTGTTCCA
TbF3H_F	AACCCCGGATCCatggctccaaccgttagc
TbF3H_R	GTTCCATGTCGACtcaggccaggatttgttc
q-TbF3H_F	GCTGATGGACCTGGCGTGTAAG
q-TbF3H_R	GAGGGCACTTTGGGTAGTAGTTCAC
C1168.2_F	GCTTGAAGATATTGTCCCCTCATCCC
C1168.2_R	AGTCATCCTTTGTGCTGCCATTCTC

**Table 2:** Promoter-specific primer sequences.

Primer Name	Sequence (5′-3′)
P_SCS2__F	gcgttattgaaaaacatgatgcacgattcctttct
P_SCS2__R	GGGAGCCATGGATCCGAATacttaggttcgcggag
P_ADE16__F	ggaatcgtgcatcattatcaagcaaacccctac
P_ADE16__R	GGAGCCATGGATCCttttagctcttttgttttttg
P_HOM6__F	ggaatcgtgcatcatgtttttcaataacgcacatg
P_HOM6__R	CCGTGGGAGCCATGGATCCtttttttttattattcgattg
P_PTC3__F	gaatcgtgcatcattaaaaagacgttatcatg
P_PTC3__R	GGAGCCATGGATCCgttatctctctctttcttc
P_TDH1__F	tgtgtgGAAACCACACCGTGGGG
P_TDH1__R	cccatGGATCCtttgttttgtgTGTAAATTTAG
P_INO1__F	GTTTTACGTGATCgaagacgatgagGCCGGTG
P_INO1__R	ggagccatGGATCCTGttacttctttttcactg

**Table 3:** Optimization of medium fermentation conditions.

Component	Concentration					
Yeast	1%	2%	3%	4%	5%	6%
Tryptone	2%	4%	6%	8%	10%	12%
Glucose	2%	4%	8%	12%	16%	20%

**Table 4:** Optimization of cofactor addition conditions.

Component	Concentration						
α-Ketoglutaric acid	0.005	0.01	0.05	0.1	0.5	1	2
FeSO_4_/mM	0.005	0.01	0.05	0.1	0.5	1	2
Ascorbic acid	0.005	0.01	0.05	0.1	0.5	1	2
CaCl_2_	0.01	0.05	0.1	0.2	0.3	0.4	0.5

## Data Availability

Data are contained within the article.
